# Time‐dependent diffusion in undulating thin fibers: Impact on axon diameter estimation

**DOI:** 10.1002/nbm.4187

**Published:** 2019-12-23

**Authors:** Jan Brabec, Samo Lasič, Markus Nilsson

**Affiliations:** ^1^ Department of Clinical Sciences Lund, Medical Radiation Physics Lund University Lund Sweden; ^2^ Random Walk Imaging AB Lund Sweden; ^3^ Department of Clinical Sciences Lund, Diagnostic Radiology Lund University Lund Sweden

**Keywords:** axon diameter, axonal trajectories, diffusion MRI, diffusion spectrum, low frequency, restricted diffusion, time dependence, undulation

## Abstract

Diffusion MRI may enable non‐invasive mapping of axonal microstructure. Most approaches infer axon diameters from effects of time‐dependent diffusion on the diffusion‐weighted MR signal by modeling axons as straight cylinders. Axons do not, however, propagate in straight trajectories, and so far the impact of the axonal trajectory on diameter estimation has been insufficiently investigated. Here, we employ a toy model of axons, which we refer to as the undulating thin fiber model, to analyze the impact of undulating trajectories on the time dependence of diffusion. We study time‐dependent diffusion in the frequency domain and characterize the diffusion spectrum by its height, width, and low‐frequency behavior (power law exponent). Results show that microscopic orientation dispersion of the thin fibers is the main parameter that determines the characteristics of the diffusion spectra. At lower frequencies (longer diffusion times), straight cylinders and undulating thin fibers can have virtually identical spectra. If the straight‐cylinder assumption is used to interpret data from undulating thin axons, the diameter is overestimated by an amount proportional to the undulation amplitude and microscopic orientation dispersion of the fibers. At higher frequencies (shorter diffusion times), spectra from cylinders and undulating thin fibers differ. The low‐frequency behavior of the spectra from the undulating thin fibers may also differ from that of cylinders, because the power law exponent of undulating fibers can reach values below 2 for experimentally relevant frequency ranges. In conclusion, we argue that the non‐straight nature of axonal trajectories should not be overlooked when analyzing and interpreting diffusion MRI data.

## Graphical abstract

We quantified characteristics of the time‐dependent diffusion in a toy model of undulating axons by analysis of the diffusion spectra. Microscopic orientation dispersion of the undulating thin fibers was the main factor determining the spectrum. At lower frequencies, the spectra were virtually identical to those from straight cylinders, which may lead to misinterpretation of undulations as cylinders.

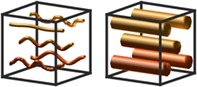

**Abbreviations**
dMRI: diffusion MRI
MSE: mean squared error
SNR: signal‐to‐noise ratio
μOD: microscopic orientation dispersion

## 1 INTRODUCTION

Tissue microstructure emerges from a finer scale and is tightly coupled with its biological function. For example, the conduction velocity through the axon is determined by its diameter, which varies in size in the central nervous system between approximately 0.1 and 15 μm.^1^, ^2^ Non‐invasive quantification of microstructural properties is appealing to study structure–function interactions, and microstructural imaging with diffusion MRI (dMRI) has emerged as a promising technology for this purpose.^3^

Quantification of the axon diameter has been the focus of many dMRI studies. Stanisz et al analyzed dMRI data from the optic nerve with a compartment model and found the estimated parameters to reflect histological counterparts.^4^ Assaf et al used *q*‐space imaging to show that mean square displacements of water molecules in the rat spinal cord are of the same order of magnitude as the axon diameter.^5^ Later work found model‐based estimates of the axon diameter distribution in the excised porcine and sciatic nerves to be in good agreement with values obtained from histology.^6^ Barazany et al reported axon diameter distributions of the rat corpus callosum estimated in vivo.^7^ Alexander et al found an axon diameter index in the human corpus callosum in vivo to be substantially higher than expected, but aligned with known trends.^8^

In all of these studies, the axon diameter mapping was based on modeling the time dependence of the diffusion within the axon. Analytical models for time‐dependent diffusion exist for simple geometries such as parallel planes, straight cylinders, and spheres,^9^, ^10^ and were thus appealing model‐building blocks. The early model by Stanisz et al represented axons as prolate ellipsoids, glial cells as spheres with partially permeable membranes, and the extra‐axonal space as having anisotropic but time‐independent (Gaussian) diffusion.^4^ Later models represented axons by straight impermeable cylinders, neglected glial cells, and retained the assumption of Gaussian diffusion in the extra‐axonal space.^6^, ^8^, ^11^ Axon diameters have been represented by a single value (CHARMED^11^ and ActiveAx^8^) or by a gamma distribution of diameters (AxCaliber^6^). Apart from the axon diameter, the models explain the signal also by other features of the tissue, such as the density of axons,^12^, ^13^, ^14^ their orientation,^15^, ^16^ and, in some cases, the geometry of the extra‐axonal space.^17^, ^18^

The assumption that axons can be modeled as straight impermeable cylinders is today widely used. However, the validity of this assumption needs examination.^18^, ^19^ For example, the diameter of an axon can vary along its length,^20^, ^21^, ^22^ and axons may feature fine morphological details such as spines, leaflets, or beads.^23^ Perhaps most important is that axons do not propagate straight.^24^ Some axons exhibit approximately sinusoidal trajectories with undulation amplitudes an order of magnitude higher than the diameter. Such undulating axons are present extra‐cranially in, for example, the phrenic nerve^25^ and in the cranial nerves, such as the root of the trigeminal nerve.^26^ Undulations also appear to be present in parts of the central nervous system, e.g. in the corona radiata, the optical nerve radiations, and the corpus callosum.^24^, ^27^ This is important because it could lead to overestimated axon diameters, unless accounted for.^24^, ^28^, ^29^

**Figure 1:**
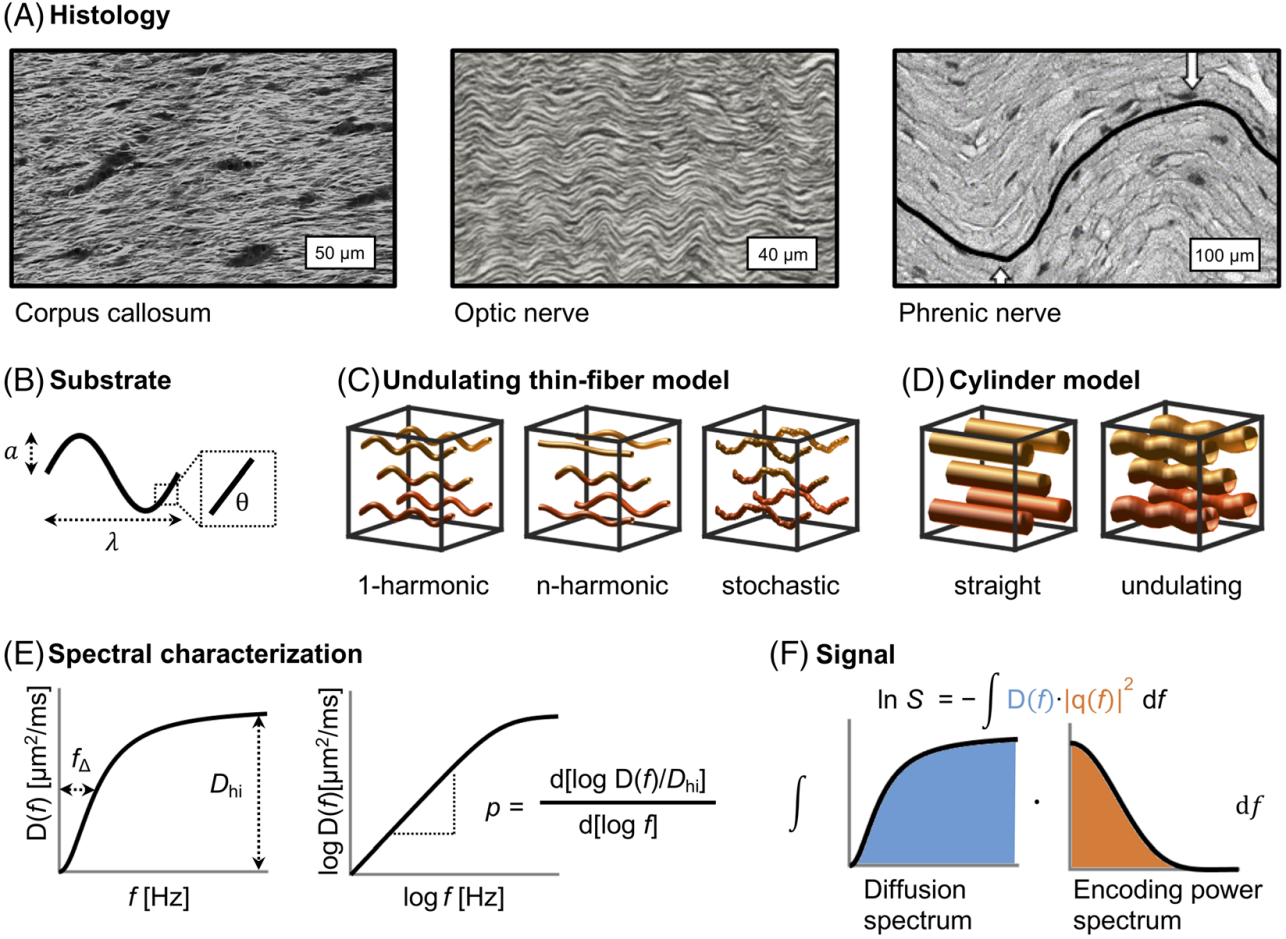
From histology to diffusion‐weighted signals. (A) Images of axons, which inspired our undulating thin fiber model. Axons in the corpus callosum,^30^ optic nerve,^31^ and phrenic nerve^25^ exhibit ubiquitously sinusoidal undulation patterns. (B) Underlying sine wave parameters used in the simulations (amplitude *a*, wavelength *λ*, and discretization into segments with variable angle θ). (C) Three cases of our undulating thin fiber model (1‐harmonic, *n*‐harmonic, and stochastic) and (D) comparison with the cylinder model (non‐dispersed straight and undulating). (E) The diffusion spectra, which were characterized in terms of their spectral width (*f*_Δ_), height (*D*_hi_), and low‐frequency behavior (*p*). (F) The connection between diffusion spectra, encoding power spectra, and the resulting signal through the first‐order cumulant expansion. Images in a were reproduced with permission from Elsevier, John Wiley & Sons, Inc., and the Institute of Electrical and Electronics Engineers (IEEE)

The purpose of this study was to determine the features of non‐straight axons that are observable with diffusion encoding schemes typically achievable with clinical MRI systems and explore how axonal undulation could lead to misinterpretation of results based on the straight‐cylinder assumption. Our investigation utilized a toy model of axons and their trajectories, which we refer to as the undulating thin fiber model. This model enabled us to study effects of non‐straight trajectories on the time dependence of intra‐axonal diffusion. Although the trajectories are complex in biological tissue, the proposed toy model was simplified to identify parameters of relevance and to capture the gross relation between the structure and the diffusion time dependence (Figure 1). The latter was characterized using the diffusion spectrum as defined by a frequency analysis of signal attenuation in the first‐order cumulant expansion, where signal attenuation is determined by the inner product of the encoding spectrum and the diffusion spectrum (Figure 1F).^9^ Our analysis focused on four topics. First, we used the undulating thin fiber model to investigate theoretically which properties of the trajectories determine the characteristics of the diffusion spectrum (Figure 1E). Second, we compared results from the theoretical approach with those from numerical simulations. Third, we compared the diffusion spectra from our fiber model with the ones arising from straight and undulating cylinders (Figure 1D)^8^ and from the short‐range disorder,^17^, ^18^ representing intra‐ and extra‐axonal diffusion, respectively. Fourth, we assessed when and why the models that assume straight cylinders overestimate the axon diameter in the presence of axonal undulations.

## 2 THEORY

We represent axons as spin‐carrying, infinitesimally thin, and infinitely long undulating fibers. Let the undulating thin fiber be given in a two‐dimensional plane by the longitudinal (*x*) and transversal (*y*) coordinates, related by

(1) $$y\left(x|a,\lambda ,\phi \right)=a\cdot \sin\left(2\pi \cdot \frac{x}{\lambda }+\phi \left(x\right)\right),\;$$

where *a* is the undulation amplitude, *λ* is the undulation wavelength, and ϕ(*x*) is a phase‐modulating factor representing stochastic variations (Figure 1B). The toy model was inspired by the histology of brain tissue (Figure 1A), which shows nearly sinusoidal axonal trajectories with undulation amplitudes an order of magnitude larger than the axon diameter.

We used the undulating thin fiber model to analyze three cases with increasing complexity, using both theoretical tools and numerical simulations (Figure 1C). The first case comprised fiber trajectories described by a single amplitude and wavelength and a constant phase‐modulating factor (1‐harmonic case). The second case comprised multiple 1‐harmonic fiber trajectories characterized by a distribution of amplitudes and wavelengths, again with a constant phase (*n*‐harmonic case). The third case comprised 1‐harmonic trajectories with a stochastic phase (the stochastic case).

### 2.1 The diffusion spectrum

Properties of the diffusion spectrum were analyzed for all three cases in the direction transversal to the main fiber direction in the two‐dimensional plane where undulating thin fibers are defined, because it is this component that contributes to dMRI‐based estimates of the axon diameter.^32^ The signal attenuation can then be analyzed in terms of two factors^9^: one that describes the diffusion in the system (the diffusion spectrum) and one that determines which parts of the diffusion spectrum are encoded into the signal (the encoding power spectrum, also known as the dephasing power spectrum). The diffusion spectrum is defined as the Fourier transform of the velocity autocorrelation function^9^

(2) $$D\left(f\right)=F\left\{〈v\left(t\right)v\left(0\right)〉\right\}=\frac{1}{2}\int_{-\infty }^{\infty }〈v\left(t\right)v\left(0\right)〉\cdot e^{-2\mathrm{i\pi}\cdot t\cdot f}\;dt.$$

The velocity autocorrelation function 〈v(*t*)v(0)〉 is in turn related to the mean square displacement 〈∆y^2^(*t*)〉 as

(3) $$〈v\left(t\right)v\left(0\right)〉=\frac{1}{2}\frac{d^{2}}{dt^{2}}〈∆y^{2}\left(t\right)〉.$$

The quantities—the mean square displacement 〈*∆y*^2^(*t*)〉, the velocity autocorrelation function 〈v(*t*)v(0)〉 and the diffusion spectrum D(*f*) provide the same information. Up to the first order the attenuation is given by the inner product of the diffusion spectrum D(*f*) and the encoding power spectrum |q(*f*)|^2^

(4) $$S\approx \exp\left(-\int_{-\infty }^{+\infty }D\left(f\right)\cdot {\left|q\left(f\right)\right|}^{2}\;df\right).$$

The encoding spectrum q(*f*) is defined from a gradient waveform g(*t*) as

(5) $$q\left(f\right)=F\left\{q\left(t\right)\right\}=F\left\{\gamma \int_{0}^{\tau }g\left(t\right)\;dt\right\}=\int_{-\infty }^{+\infty }q\left(t\right)\;e^{-2\mathrm{\pi i}\cdot t\cdot f}\;dt,\;$$

where *τ* is the echo time. For completeness, note that the *b*‐value is the total encoding power given by

(6) $$b=\int_{-\infty }^{+\infty }{\left|q\left(f\right)\right|}^{2}\;df.$$

### 2.2 Characterizing the diffusion spectrum

Our first aim was to predict features of the diffusion spectra from the parameters of the undulating thin fiber model. Corresponding features of the diffusion spectrum for a straight cylinder were used for reference. Four spectral features were considered: the spectral width (*f*_∆_), the spectral height (*D*_hi_), the spectral shape, and the low‐frequency behavior in terms of the power law exponent (*p*) (Figure 1E). An additional feature was also considered: the diffusivity at zero frequency (infinite diffusion times). However, this was trivially zero because the maximal mean square displacement is bounded by the outermost positions of the fiber trajectories in our fiber model. In the presence of orientation dispersion of the main fiber directions of different fibers, which we refer to as a macroscopic orientation dispersion, this would not hold true, but we limited our analysis to the case without macroscopic orientation dispersion.

#### 2.2.1 Spectral height

The high‐frequency limit *D*_hi_ represents diffusivity at shorter diffusion times (higher frequencies), when the diffusion is unrestricted (Figure 1E). In this regime, the displacements of diffusing particles become small enough that they probe only their immediate surroundings. For cylinders, *D*_hi_ is trivially given by the bulk diffusivity *D*_0_. For all cases of our fiber model, *D*_hi_ can be obtained by noting that in the limit of shorter diffusion times we can approximate an undulating thin fiber by straight but orientationally dispersed and disconnected segments. The diffusion coefficient transversal to an angulated segment is given by

(7) $$D\left(t\to 0\right)=〈\sin^{2}\left(\theta \left(x\right)\right)〉\cdot D_{0},\;$$

where *x* is the position of the segment, 〈·〉 denotes averaging over all segments, and θ is the angle between the direction of the segment and the main fiber direction. We define the microscopic orientation dispersion (μOD) as

(8) $$\mathrm{\mu OD}=〈\sin^{2}\left(\theta \left(x\right)\right)〉.$$

Examples of μOD values are shown in Table 1. Note that we define μOD as a measure of dispersion within individual fibers and macroscopic orientation dispersion as a dispersion of the collection of fibers (namely their main fiber direction). For all three cases, we can thus predict that

(9) $$D_{\mathrm{hi}}=\mathrm{\mu OD}\cdot D_{0}.$$

Note that this would imply that *D*_hi_ < *D*_0_ also for infinite frequencies (eg the diffusion is never free), which indicates the limit of our toy model. That is why we denote the spectral height by the symbol *D*_hi_ and not by, eg, *D*_∞_. The asymptotic behavior limits the scope of our fiber model, which we will bring up again in the discussion.

**Table 1:** μOD values corresponding to 1‐harmonic axonal trajectories. One μOD value (Equation 8) can correspond to more pairs of undulation amplitudes and wavelengths

		Wavelength *λ* [μm]				
		10	20	30	40	50
Amplitude *a* [μm]	1	0.16	0.05	0.02	0.01	0.008
	2	0.41	0.16	0.08	0.05	0.03
	3	0.59	0.29	0.16	0.10	0.07

For the *n*‐harmonic case, *D*_hi_ is given by the average of contributions from *n* different 1‐harmonic fibers

(10) $$D_{\mathrm{hi}}=〈D_{\mathrm{hi};\;i}〉\approx D_{0}\cdot 〈{\mathrm{\mu OD}}_{i}〉.$$

#### 2.2.2 Spectral width

The spectral width captures the frequency where the time‐dependent diffusion effects have the highest impact on the signal. It is the frequency corresponding to half of the highest diffusivity (half width at half maximum of the diffusion spectrum, Figure 1E). For cylinders, the spectral width (*f*_*∆*_) is inversely related to the time required for the mean square displacement (〈*∆y*^2^(*t*)〉 = 2*D*_0_*t*) to approach the fiber diameter (*d*)

(11) $$f_{∆}=k_{c}\cdot \frac{D_{0}}{d^{2}}\;\left[\mathrm{Hz}\right],\;$$

where $k_{c}=\sqrt{1536/7\cdot 1/4\pi^{2}}\approx 2.35$ is a proportionality constant determined by analyzing the infinite sum of the analytical diffusion spectrum for cylinders.^9^, ^17^ The spectral width is thus an important parameter in the cylinder case because it is directly related to the cylinder diameter.

For the undulating thin fiber model, we assess each case separately. For the 1‐harmonic case, the diffusion process is similar to that in the cylinder because the outer limits of the fiber trajectory will act as reflecting boundaries. However, longer diffusion times are needed to reach the boundaries as the water molecules cannot diffuse along the shortest paths but are bounded by the fiber trajectory. We thus postulate that the spectral width is

(12) $$f_{∆}\approx k_{h}\cdot \frac{{\tilde{D}}_{0}}{a^{2}},\;$$

where *k*_h_ is a proportionality constant and ${\tilde{D}}_{0}$ is the apparent bulk diffusion coefficient. We did not find an analytical expression for *k*_h_, but determined it from the numerical simulations. The bulk diffusivity coefficient *D*_0_ is reduced by the path length ratio between straight and undulating fibers:

(13) $${\tilde{D}}_{0}=D_{0}\cdot \frac{a^{2}}{{\left(\int_{C}\;y\left(x\right)\;dx\right)}^{2}\;},$$

where the denominator denotes a path integral over one wavelength of the sine wave. The path length ratio can be linked to μOD:

(14) $$\frac{a^{2}}{{\left(\int_{C}\;y\left(x\right)\;dx\right)}^{2}\;}=\frac{{〈\left|\mathrm{dy}\left(x\right)\right|〉}^{2}}{{〈dl〉}^{2}}=〈\frac{{\mathrm{dy}}^{2}\left(x\right)}{dl^{2}}〉=〈\sin^{2}\left(\theta \left(x\right)\right)〉=\mathrm{\mu OD},\;$$

where d*l* is the length of a segment (d*l*^2^ = d*x*^2^(*x*)+d*y*^2^(*x*)), which is in our case kept constant. Taken together, we predict that the spectral width for the 1‐harmonic case is given by

(15) $$f_{∆}\approx k_{h}\cdot \frac{D_{0}}{a^{2}}\cdot \mathrm{\mu OD}.$$

For the *n*‐harmonic case, we first note that fibers with higher *D*_hi_ have a larger impact on the spectral width *f*_*∆*_. The width can thus be approximated by a weighted average of the 1‐harmonic spectral widths *f*_*∆*;*i*_, where the weight *w*_*i*_ is given by the spectral height *D*_hi;*i*_, according to

(16) $$f_{∆}\approx \frac{〈w_{i}\cdot f_{∆;\;i}〉}{〈w〉}=\frac{〈D_{\mathrm{hi};\;i}\cdot f_{∆;\;i}〉}{〈D_{\mathrm{hi};\;i}〉}=k_{h}\cdot D_{0}\cdot \frac{〈{\mathrm{\mu OD}}_{i}^{2}/a_{i}^{2}〉}{〈{\mathrm{\mu OD}}_{i}〉}.$$

The stochastic case can be analyzed by using the diffusion spectra (Equation 2), similarly to the *n*‐harmonic case (Equation 16), by assuming each segment of the stochastically undulating fiber to have its own spectral height and width according to Equations 9 and 15, but using the local μOD and amplitude, which is given by the maximal deviation from the straight path (*a*_max_). The local μOD was defined as μOD(*x*) = sin^2^(*θ*(*x*)). The spectral width can then be approximated by averaging all local segments using the same approach as in Equation 16,

(17) $$f_{∆}\approx k_{s}\cdot \frac{{\tilde{D}}_{0}}{{a_{\max}}^{2}}=k_{s}\cdot \frac{D_{0}}{{a_{\max}}^{2}}\cdot \frac{〈{\mathrm{\mu OD}}^{2}\left(x\right)〉}{\mathrm{\mu OD}}$$

where *k*_s_ is a proportionality constant that can be determined from simulations, ${\tilde{D}}_{0}$ is given by

(18) $${\tilde{D}}_{0}=D_{0}\cdot \frac{〈{\mathrm{\mu OD}}^{2}\left(x\right)〉}{〈\mathrm{\mu OD}\left(x\right)〉}\;$$

and 〈μOD(*x*)〉 = μOD.

#### 2.2.3 Simplified spectral shape and low‐frequency behavior

To analyze the shape of the diffusion spectrum, we represent it by an approximation and study the differences between the simplified and simulated spectra. For simple geometries such as parallel planes, straight cylinders, and spheres, the diffusion spectra are given analytically by an infinite sum of Lorentzians^9^, ^33^,

(19) $$D\left(f\right)=\sum_{k=1}^{\infty }D_{\mathrm{hi};\;k}\cdot \frac{f^{2}}{{f_{∆;\;k}}^{2}+f^{2}}=D_{0}\sum_{k=1}^{\infty }a_{k}B_{k}\cdot \frac{f^{2}}{{\left(a_{k}D_{0}\right)}^{2}+f^{2}},\;$$

where *D*_hi;*k*_ and *f*_*∆*;*k*_ are the height and width of the *k*th Lorentzian contribution, respectively, and *a*_*k*_ and *B*_*k*_ are coefficients related to the geometry

(20) $$a_{k}={\left(\frac{\zeta_{k}}{r}\right)}^{2}\text{and}\;B_{k}=\frac{2\left(r/\zeta_{k}\right)}{{\zeta_{k}}^{2}+1-\dim}.$$

Here 2*r* is the diameter and ζ_*k*_ are the kernels of

(21) $$\zeta J_{\dim/2-1}\left(\zeta \right)-\left(\dim-1\right)J_{\dim/2}\left(\zeta \right)=0,\;$$

where *J*_*υ*_ denotes the *υ*th‐order Bessel function of the first kind, and dim = i.e. 1, 2, or 3 for planar, cylindrical, or spherical restrictions, respectively. Although Equation 19 contains an infinite sum, we note that the majority of the spectrum is accounted for by the first Lorentzian term, given by the coefficient *a*_1_*B*_1_, which is 0.83 for cylinders. A simplified spectrum can thus be defined by a single‐Lorentzian approximation L(*f*),

(22) $$D\left(f\right)\approx L\left(f\right)=D_{\mathrm{hi}}\cdot \frac{f^{2}}{{f_{∆}}^{2}+f^{2}},\;$$

where *D*_hi_ and *f*_*∆*_ are the two features determining to the spectral height and width, respectively. This simplified spectrum will be used to study dissimilarities between straight cylinders and the undulating thin fiber model.

Another aspect of the simplification of the spectra is its low‐frequency behavior, which contains information on the gradual coarse‐graining process over structural length‐scales.^34^, ^35^, ^36^, ^37^ The single‐Lorentzian approximation (Equation 22) can be expanded to the second order as

(23) $$L\left(f\right)\approx \frac{D_{\mathrm{hi}}}{{f_{∆}}^{2}}\cdot f^{2}.$$

Plugging in the results for the cylinder case (*D*_hi_ = *D*_0_ and Equation 11) yields

(24) $$L\left(f\right)\approx \frac{1}{k_{c}^{2}}\cdot \frac{d^{4}}{D_{0}}\cdot f^{2}\;\left[\mathrm{Hz}\right]\;$$

and in the 1‐harmonic case (Equations 9 and 15)

(25) $$L\left(f\right)\approx \frac{1}{k_{h}^{2}}\cdot \frac{a^{4}}{\mathrm{\mu OD}\cdot D_{0}}\cdot f^{2}\;\left[\mathrm{Hz}\right].$$

Under the assumption that the single‐Lorentzian approximation describes well the spectra for the undulating thin fiber model, the parameters that determine the low‐frequency behavior are *a* and μOD. The diameter thus relates to the parameters in the 1‐harmonic case of our fiber model as

(26) $$d\approx \sqrt{\frac{k_{c}}{k_{h}}}\cdot \frac{a}{\sqrt[{4}]{\;\mathrm{\mu OD}}}.$$

This relation suggests that undulations due to non‐zero μOD can bias diameter estimations. Note that Equation 26 assumes that the low‐frequency second‐order expansion (Equation 23) is an adequate simplification of the diffusion spectrum in the range of frequencies attainable in typical dMRI experiments, which is not guaranteed. We thus also investigated the low‐frequency behavior by simulations.

## 3 METHODS

Numerical simulations were performed to test the theoretical predictions. Diffusion spectra were generated for the 1‐harmonic and stochastic cases by computing mean square displacements 〈*∆y*^2^(*t*)〉 and from these the velocity autocorrelation functions 〈v(*t*)v(0)〉 using Equation 3. Finally, 〈v(*t*)v(0)〉 was Fourier‐transformed (Equation 2) to obtain the diffusion spectrum *D*(*f*). Spectra of the *n*‐harmonic case were generated by averaging the spectra from the 1‐harmonic case (for details, see the AppendixA). The analysis was implemented in MATLAB (MathWorks, Natick, MA, USA) and is available at https://github.com/jan-brabec/undulating_fibers.

### 3.1 Substrate definition

The parameters of the undulating thin fiber model that were used in the simulations are listed in Table 2, and were selected to represent a range of conditions relevant for the corpus callosum by observing the histology images shown in Figure 1A. The pathways defined by our fiber model were discretized into straight segments with equal lengths of d*l* = 0.1 μm. We consider the analysis only for mild undulations. For the 1‐harmonic case, we investigated undulation amplitudes of *a* = 1, 2, and 3 μm and wavelengths of *λ* = 10, 20, 30, 40, and 50 μm. The *n*‐harmonic case was generated from a gamma distribution of amplitudes and wavelengths, which is described in the appendix. For the stochastic case, variations were introduced into the harmonic sine waves by modeling the phase‐modulating factor ϕ(*x*) in Equation 1 as a first‐order autoregressive process, AR(1): ϕ(*x*_*i*_) = *ρx*_*i*− 1_+*ς*(*x*), where *ς*(*x*) are independent normally distributed random numbers. The randomly generated numbers were normalized and cumulatively summed. To avoid high‐frequency fiber fluctuations that would represent unphysical turns of the fiber, the random numbers were smoothed using a moving average filter with a width of 0.5 μm.

**Table 2:** Estimated undulating parameters from histology. Estimated values of undulation amplitude *a*, wavelength *λ*, and mean axon diameter *d* of optic nerve,^31^, ^38^ corpus callosum,^1^, ^39^, ^40^, ^41^, ^42^ and phrenic nerve.^25^, ^43^ The axon diameters in corpus callosum are in the range 0.5–15 μm and their volume‐weighted average is below 1 μm

	Optic nerve	Corpus callosum	Phrenic nerve
*a* [μm]	1–20	1–10	20–100
*λ* [μm]	10–50	10–100	100–1000
*d* [μm]	1	0.5	4–5

### 3.2 Numerical simulations

#### 3.2.1 Gaussian sampling

To estimate the mean square displacement, we implemented what we refer to as the Gaussian sampling method. It assumes one‐dimensional Gaussian diffusion along the fiber trajectory. Displacements were thus described with a normal distribution with a mean of zero and a variance of *σ*^2^ = 2*D*_0_*t*, with the bulk diffusion coefficient set to *D*_0_ = 1.7 μm^2^/ms. The mean squared displacements transversal to the main fiber direction 〈*∆y*^2^(*t*)〉 were then computed using the algorithm described by pseudo‐code in the Appendix.

### 3.3 Validation of diffusion spectra and signal simulations

The Gaussian sampling approach was verified against an alternative approach using Monte Carlo simulations. 10^6^ particles were simulated as freely diffusing along a straight one‐dimensional fiber but the positions were then remapped to *x* and *y* coordinates using Equation 1. The same fiber trajectories and discretization parameters as used with the Gaussian sampling method were used in the Monte Carlo simulations, apart for a higher temporal resolution (d*t* = 10 μs).

The signal generated by the Gaussian sampling method (via Equation 4 and the simulated diffusion spectra) was also verified against that of Monte Carlo simulations, obtained by accumulating the phase (Φ_*k*_) for each particle *k* according to

(27) $$\Phi_{k}\left(t\right)=\gamma \cdot \int_{0}^{t}g\left(t'\right)\cdot x_{k}\left(t'\right)\;dt'$$

yielding attenuation as

(28) $$S=〈\exp\left(‐i\cdot \Phi_{k}\right)〉.$$

Gradient waveforms g*(t)* for the protocols by Alexander et al^8^ were used (see Table 3).

**Table 3:** Parameters of the pulsed diffusion gradients. Protocol from Alexander et al^8^

	|*G*| [mT/m]	δ [ms]	Δ [ms]	b [ms/μm^2^]
1st	58	12	80	0.50
2nd	46	15	77	0.68
3rd	57	5	87	2.45
4th	60	13	20	2.64

### 3.4 Investigation of limitations of undulating thin fiber model

To test the extent to which the undulating thin fiber model faithfully represents diffusion in undulating cylinders, simulations were also performed in the undulating cylinders using methods from Nilsson et al.^24^ We considered the mildest undulation patterns (*a* = 1 μm, λ = 50 μm; *a*/λ = 2 %) for cylinders with diameters *d* = 1, 2, 3, 5, and 10 μm. In the case of *d* = 1, 2, 3 μm one set of simulation parameters was used (d*x* = 0.02 μm, *t*_max_ = 1 s, d*t* = 2 μs, 10^6^ particles), while in the case of *d* = 5, 10 μm another set was used (d*x* = 0.1 μm, *t*_max_ = 10 s, d*t* = 50 μs, 3·10^6^ particles). Moreover, we also investigated the limitations of the first‐order approximation in Equation 4 by generating signal curves for *b*‐values up to 50 ms/μm^2^.

### 3.5 Characterization of the diffusion spectrum

Four features of the diffusion spectra were characterized and compared: spectral height, width, simplified spectral shape, and low‐frequency behavior.

#### 3.5.1 Spectral height and width

Spectral heights and widths were predicted from the parameters of our fiber model by Equations 9 and 15 in the 1‐harmonic case, Equations 10 and 16 in the *n*‐harmonic case, and Equations 9 and 17 in the stochastic case, respectively, and compared with the corresponding values estimated from the simulated spectra. Spectral widths for the 1‐harmonic (Equation 15) and stochastic (Equation 17) cases were first predicted assuming *k*_h_ = *k*_s_ = 1; the obtained data were fitted with a first‐degree polynomial and adjusted. Spectral heights were estimated from the simulated diffusion spectra as the average of D(*f*) between 900 and 1000 Hz.

Spectral widths were estimated as the frequency corresponding to half of the spectral height, which is the highest diffusivity reached in our simulation setup:

(29) $$D\left(f_{∆}\right)=\frac{1}{2}D_{\mathrm{hi}}.$$

#### 3.5.2 Simplified spectral shape

Simulated spectra were quantified by fitting the two free parameters (height and width) of the single‐Lorentzian approximation (Equation 22). To test whether this simplified representation generated signals significantly different from the actual simulated spectra, we compared signals generated from the simulated spectra with those from the fitted single‐Lorentzian spectra. Pure noise at levels expected at clinical scanners (signal‐to‐noise ratio, SNR ≈ 50) would generate a mean square error of the signal of MSE = 4 · 10^−4^ (see the Appendix for details). If the MSE for signals generated by the two approaches is lower than that expected from noise alone, the differences are indistinguishable. Four simulation setups were investigated: undulating thin fibers from the 1‐harmonic case (*a* = 2 μm, *λ* = 30 μm), the *n*‐harmonic case, and the stochastic case, as well as cylinders with diameter *d* = 10 μm. All cases were chosen so that they had a similar spectral width and height (1‐harmonic, 10 Hz and 0.13 μm^2^/ms; *n*‐harmonic, 8 Hz and 0.14 μm^2^/ms; stochastic, 7 Hz and 0.13 μm^2^/ms; cylinders, 11 Hz but 1.7 μm^2^/ms).

### 3.6 Low‐frequency behavior

The low‐frequency part of the spectrum can be approximated by *cf*^*p*^, where *c* is a normalization constant and the value of exponent *p* can reveal structural universality classes.^34^, ^36^, ^37^ We studied the low‐frequency behavior by estimating the exponents *p* by two different methodologies: first by fitting (*p*_fit_), because it is a methodology similar to other works in the field,^17^ and second by inspecting the derivative of the logarithm diffusion spectra (*p*_derivative_, Figure 1E) to assess the variability of this parameter at different frequencies. During the fitting, we compared the low‐frequency region [0 Hz; min(0.5.ċ *D*_hi_; 20 Hz)] of the simulated diffusion spectra, where the spectra can be approximated by the first‐degree polynomial

(30) $$D\left(f\right)\approx c\cdot f^{p_{\mathrm{fit}}}$$

where *p*_fit_ > 0 is a real number.

In the second case, the exponent *p*_derivative_ was computed as the derivative of the diffusion spectrum normalized to *D*_hi_:

(31) $$p_{\text{derivative}}=\frac{d\left[\log\left(D\left(f\right)/D_{\mathrm{hi}}\right)\right]}{d\left[\log f\right]}.$$

### 3.7 Implications for axon diameter mapping

Finally, we studied the link between the parameters of the undulating thin fiber model and straight‐cylinder diameters estimated by the ActiveAx model.^8^ Simulated diffusion spectra of the 1‐harmonic case were used to generate signal data based on the protocol of Alexander et al,^8^ assuming the absence of noise (infinite SNR). The ActiveAx model was constrained to the case of intra‐axonal diffusion and radial only, and the diameters were estimated by fitting the simulated signal. The estimated diameters were plotted against parameters characterizing our fiber model: undulation amplitude *a* and Equation 26 relating the diameter *d* to *a* and μOD based on the second‐order Taylor expansion.

## 4 RESULTS

### 4.1 Validation of the Gaussian sampling method

**Figure 2:**
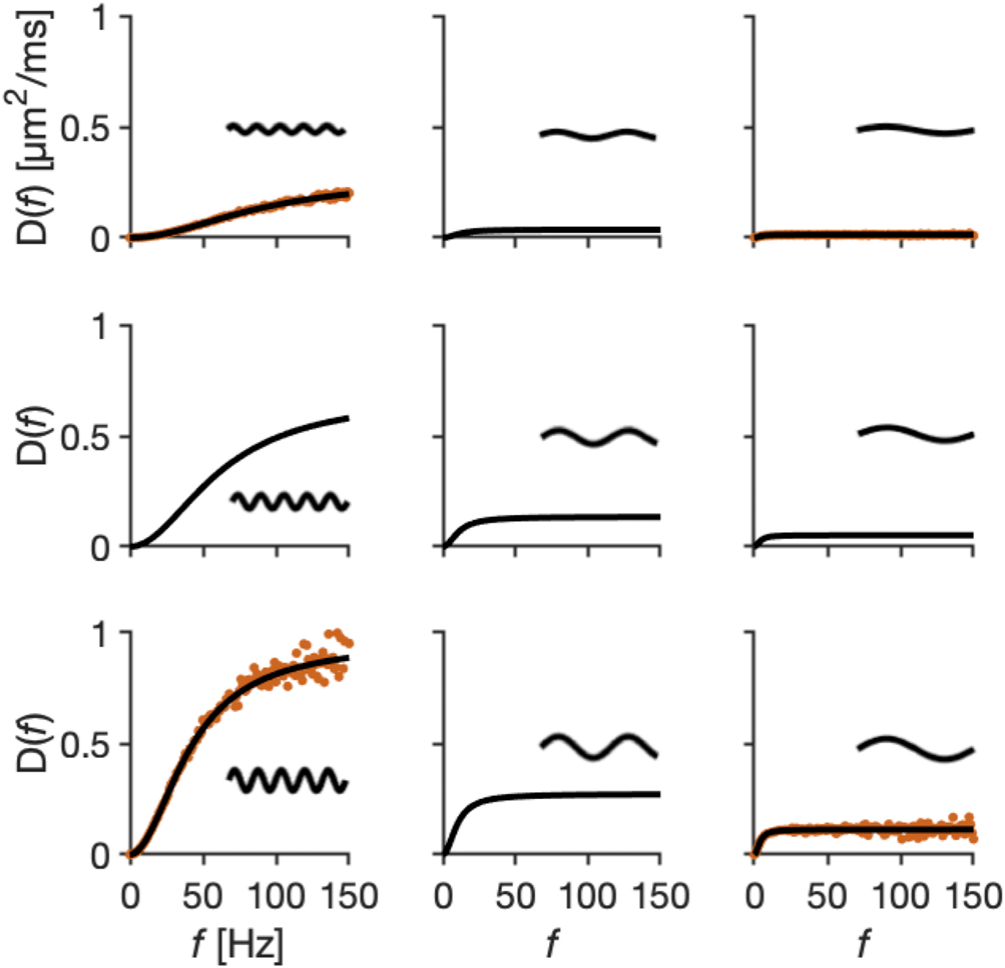
Comparisons of the Gaussian sampling method and Monte Carlo simulations. Diffusion spectra of the 1‐harmonic case characterized by wavelengths *λ* = 10, 30, 50 μm (from left to right) and amplitudes *a* = 1, 2, 3 μm (from top to bottom) computed by the Gaussian sampling method (black lines) and Monte Carlo simulations (red points). The methods agreed well, although the Monte Carlo results (red points) exhibited more noise and required more computational time than the Gaussian sampling method (black lines). This figure indicates that there is no simple relation between the spectral height or spectral width and undulation amplitude or wavelength, which means that a different parameterization of the thin fibers, such as the μOD, may be useful

Comparisons between the proposed Gaussian sampling method and the thin fiber Monte Carlo simulations showed a high agreement between the methods, but the Gaussian sampling offered a superior computational speed (2 days for Monte Carlo compared with 2 minutes for Gaussian sampling for the same discretization and fiber settings). The simulated diffusion spectra for the two methods overlapped (Figure 2), although the ones obtained by Monte Carlo simulations showed more noise, despite using one million particles in the simulation and ten times higher temporal resolution. The two methods also showed high agreement for the simulated signal attenuations (not shown as a figure), even though quite different methods were used to compute the signal (via the diffusion spectrum in Equation 4 for the Gaussian method and via simulated phase distributions with Equation 28 for the Monte Carlo method).

### 4.2 Characterizing the diffusion spectrum

**Figure 3:**
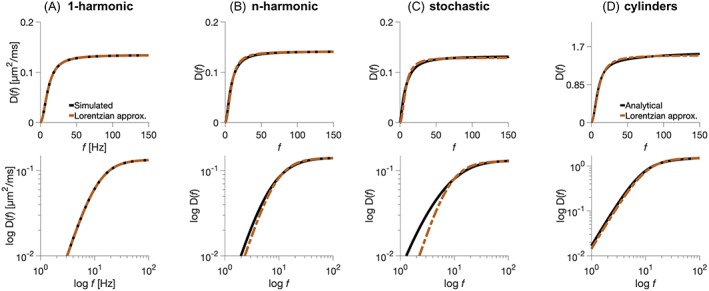
A study of the undulating thin fiber model: diffusion spectrum examples. The exact (solid black line) and simplified by the single‐Lorentzian approximation (dashed red line) diffusion spectra for the 1‐harmonic case (A), the *n*‐harmonic case (B), and the stochastic case (C) of our thin fiber model were compared with those of cylinders (D) with diameter *d* = 10 μm. The bottom row shows the same spectra as the top row but in a log–log plot. The simplified spectra deviate from the simulated ones in the low‐frequency range for the *n*‐harmonic and stochastic cases; however, in these examples, the differences are not detectable within experimental limitations when SNR ≤ 50 (Table 4)

Predicted and estimated spectral width and height agreed well and, in given examples, other features were not detectable if SNR ≤ 50 and when using the encoding protocol shown in Table 3. Figure 3 shows diffusion spectra for the 1‐harmonic, *n*‐harmonic, and stochastic cases of the thin fiber model and for a cylinder, in linear plots (top row) and log–log plots (bottom row). These examples were selected to have similar spectral heights and widths to study whether the spectra differ in some other way. All of the simulated spectra were well described by the single‐Lorentzian approximation L*(f)* (Equation 22), at least qualitatively. Quantitatively, the signals generated from either the simulated or the simplified Lorentzian spectra were highly similar, with a difference indistinguishable from that induced by noise, for SNR of 50 (Table 4), with the 1‐harmonic case being the closest to the simplified spectrum. This means that in the examples investigated the differences between the simulated and simplified spectra cannot be experimentally distinguished under realistic noise levels when using the protocol in Table 3. The simplified spectra (Equation 22) are defined by only two parameters, corresponding to spectral width and height.

**Table 4:** Comparison of mean square errors (MSE) of the simulated and simplified spectra by the single‐Lorentzian approximations (Equation 22). Signals were generated from simulated diffusion spectra corresponding to cylinders and three examples of different cases with similar spectral width and height. They were fitted by a single‐Lorentzian approximation (Figure 3; Equation 22). The smallest error is found in the example of the 1‐harmonic case, the largest for a cylinder, but all values are in these examples below the MSE obtained for noise (4 · 10^−4^) at SNR = 50

	Cylinders	1‐harmonic	*n*‐harmonic	Stochastic
MSE of S(*f*)	3.1 · 10^−4^	1.8 · 10^−8^	1.3 · 10^−4^	2.3 · 10^−4^

**Figure 4:**
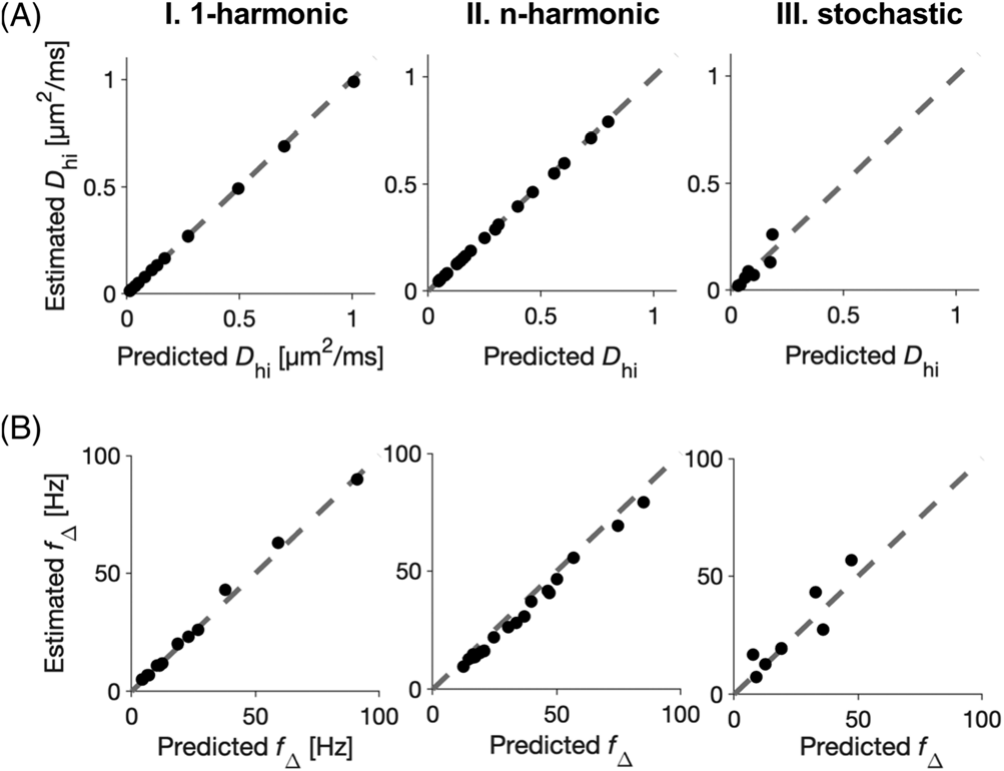
A study of the undulating thin fiber model: Prediction of diffusion spectra parameters. (A) Predicted and estimated spectral heights *D*_hi_ are aligned for the 1‐harmonic case (I), the *n*‐harmonic case (II), and the stochastic case (III) of the undulating thin fiber model. Values of *D*_hi_ were predicted using Equation 9 for the 1‐harmonic and stochastic cases and using Equation 10 for the *n*‐harmonic case. (B) Predicted and estimated spectral widths *f*_*∆*_ are aligned again for the 1‐harmonic case (I), the *n*‐harmonic case (II), and the stochastic case (III). Values of *f*_*∆*_ were predicted using Equation 15 for the 1‐harmonic case, Equation 16 for the *n*‐harmonic case, and Equation 17 for the stochastic case

Figure 4A shows spectral heights estimated from Gaussian sampling simulations versus the theoretically predicted values (Equations 9 and 10). The estimated and predicted spectral heights showed strong agreement (correlation coefficients in the 1‐harmonic and *n*‐harmonic cases above 0.99 but in the stochastic case 0.9), which confirmed that the spectral height is determined by the μOD. Figure 4B shows the corresponding analysis for the spectral width, with the theoretical predictions according to Equation 15 for the 1‐harmonic case, Equation 16 for the *n*‐harmonic case, and Equation 17 for the stochastic case of our fiber model. Proportionality constants were found to be *k*_h_ ≈ 0.34 for the 1‐harmonic and *n*‐harmonic cases and *k*_s_ ≈ 0.13 for the stochastic case. Estimated values agreed with theoretical predictions after adjustment with this proportionality constant; however, the residual variance was larger for the stochastic case compared with the other cases (correlation coefficients in the 1‐harmonic and *n*‐harmonic cases above 0.99 but in the stochastic case 0.92). The generation of the *n*‐harmonic case diffusion spectra from the synthetic 1‐harmonic diffusion spectra was enabled due to close alignment of the single‐Lorentzian approximation with the simulated spectra (Figure 3; Table 4) and their estimated spectral heights and widths with the theory (Figure 4).

### 4.3 Investigation of limitations of the undulating thin fiber model

**Figure 5:**
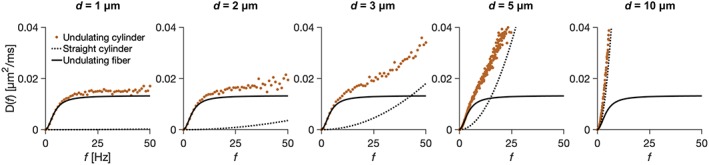
Diffusion spectra of undulating thin fibers and undulating cylinders. Plots show diffusion spectra of undulating cylinders, undulating fibers, and straight cylinders. When *d* = 1 μm and 2 μm, the spectra of undulating thin fibers closely resemble those of undulating cylinders. For *d* = 3 μm and 5 μm, the spectra start to deviate above 10 Hz. For *d* = 10 μm, the spectrum of an undulating cylinder resembles that of a straight cylinder

The spectra and signal of undulating thin fibers and undulating cylinders were similar, at least for diameters below 2–3 μm and long enough diffusion times (encoding power below 50 Hz) (Figure 5). The diffusion spectra of the 1‐harmonic undulating thin fibers were compared with undulating cylinders with the same undulation parameters, showing the spectra to agree well for diameters of *d* = 1 and 2 μm but deviate at frequencies above 10 Hz for *d* = 3 and 5 μm, although at lower frequencies they still resembled the spectra of undulating thin fibers. The spectra of undulating cylinders with *d* = 10 μm resembled those of straight cylinders.

**Figure 6:**
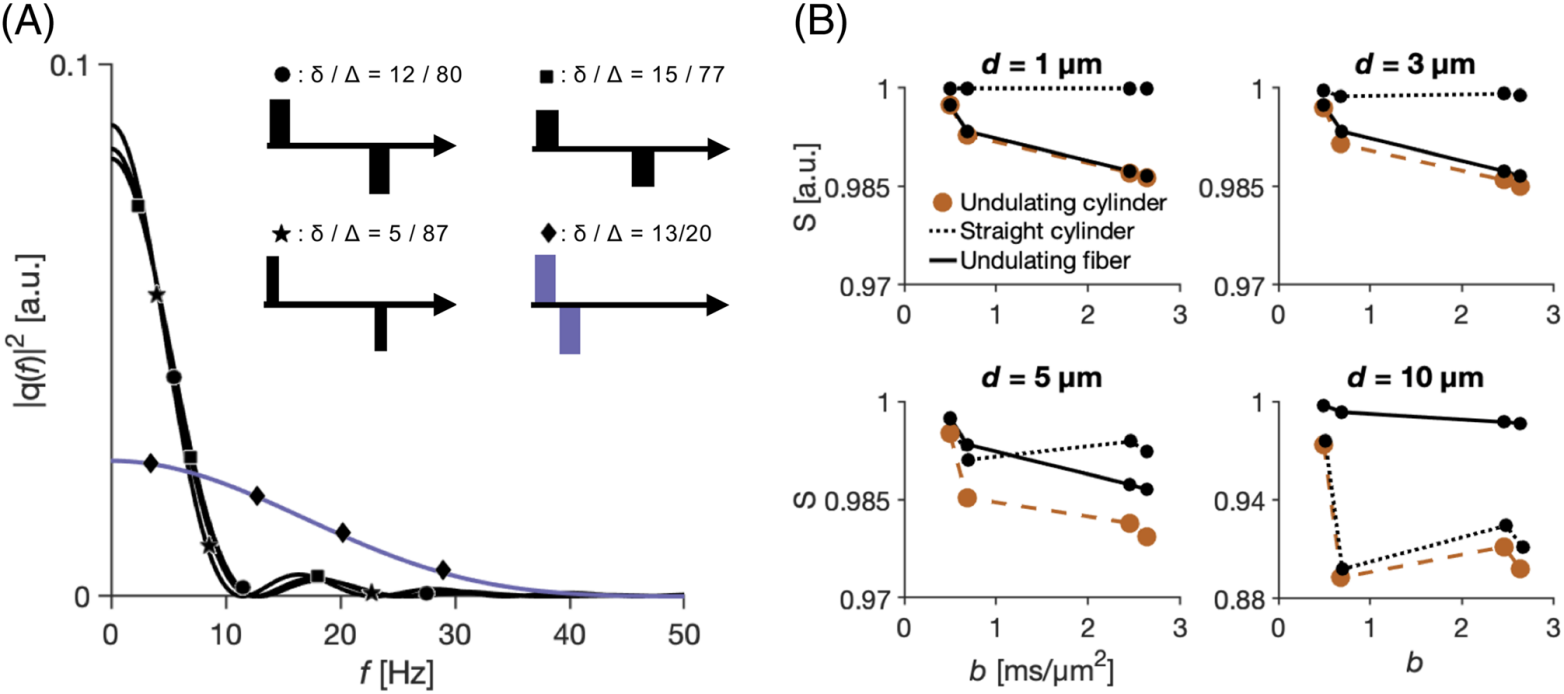
Limitations of undulating thin fiber model: Short diffusion times. (A) Gradient waveforms and their corresponding encoding spectra for the protocol by Alexander et al^8^ (Table 3). The four gradient waveforms probe only two frequency regions, [0, 10 Hz] and [0, 50 Hz]. (B) The signals of undulating cylinders (generated by gradients from a and from diffusion spectra from Figure 5) are explained well by undulating thin fibers of the same undulations when *d* = 1 μm and 3 μm. They start to deviate when *d* = 5 μm, while at 10 μm the signal from undulating cylinders is nearly identical to that from a straight cylinder. Note that we show the mildest undulation patterns (*a*/λ = 2 %)

For the protocol optimized in reference 8 and employed in this study, three of the gradient waveforms have encoding widths of 6 Hz and one of 20 Hz (Figure 6A). None of them have significant encoding power above 50 Hz (Figure 6A, black and purple curves, respectively). The signal generated by these gradient waveforms in undulating cylinders was similar to those from undulating thin fibers for *d* = 1, 2, and 3 μm (Figure 6B), but deviated and resembled the signal of straight cylinders for *d* = 10 μm.

**Figure 7:**
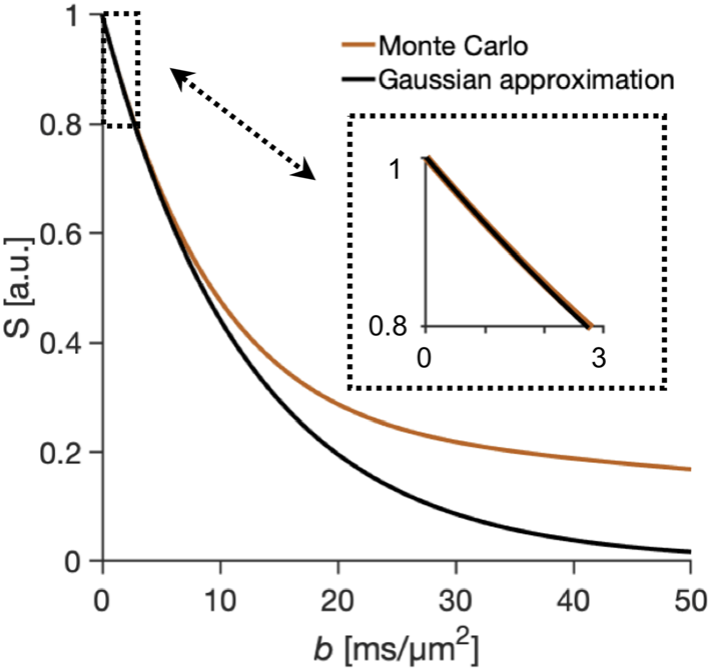
High *b*‐values. Comparison between signal obtained from Monte Carlo simulation (Equation 28) and from the first‐order approximation (Equation 4). The first‐order approximation is here shown to be valid up to attenuations of approximately 60 %. The maximal *b*‐values reached in this study were 3 ms/μm^2^ and the maximal signal attenuation 20 % (upper right box)

Finally, we note that the Gaussian approximation of the cumulant expansion (Equation 4) yields identical results to the Monte Carlo simulations of undulating thin fibers for signal attenuations up to 60 % (Figure 7),^44^ which in the case with strongest attenuations investigated means that the spectral approach is valid within practically achievable *b*‐values up to 10 ms/μm^2^.

### 4.4 Implications for study of the low‐frequency behavior

**Figure 8:**
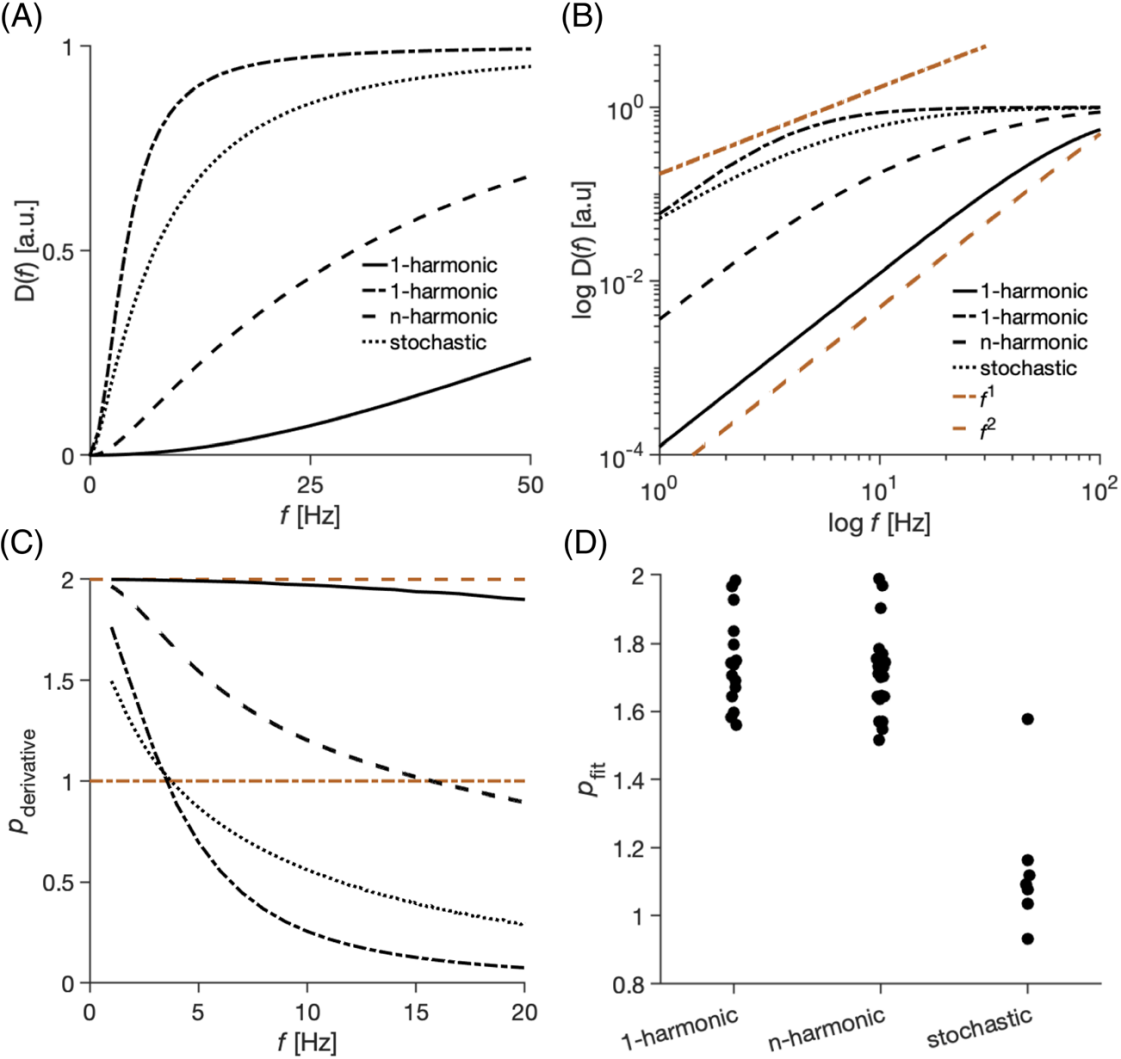
Implications for the study of the low‐frequency behavior: Estimation of exponent *p*. (A) Examples of diffusion spectra normalized to the same spectral height. (B) The same data but in a log–log plot. The curves of the 1‐harmonic case (solid and dot‐dashed black line) are aligned with the quadratic frequency curve (dashed red line), whereas the slope of the curves from the *n*‐harmonic approach the quadratic frequency curve more slowly. The stochastic case (dotted black line) is more aligned with the linear curve (dot‐dashed red line). (C) The exponent *p* (obtained via Equation 31) is not constant with respect to the frequency. (D) A distribution of exponents *p* for the simulated cases in the low‐frequency region up to 20 Hz. *n*‐harmonic fibers had gamma distributed undulation amplitudes and wavelengths further restricted to the range validated by numerical simulations (1 μm ≤ *a* ≤ 3 μm; 10 μm ≤ *λ* ≤ 50 μm)

The estimated power law exponents *p* of undulating thin fibers depend on the frequency region where they are estimated and can yield values below 2. Figure 8 highlights differences in the low‐frequency regime between the spectra for the 1‐harmonic, *n*‐harmonic, and stochastic cases. Figure 8A shows that the diffusion spectra can approach zero frequency differently both within two examples of the 1‐harmonic case (within a single case) and among different cases. Figure 8B suggests that all examples tend to resemble the low‐frequency behavior of cylinders (*p* = 2) at low enough frequencies, although the *n*‐harmonic and stochastic cases approach this limit more slowly. Figure 8C emphasizes that the estimated exponent *p* (defined by Equation 31) is not constant and depends on the frequency region. Fitting the diffusion spectra in the frequency range up to 20 Hz yielded values of the exponents *p* (defined by Equation 30) in the 1‐harmonic case from 1.6 to 2 with median 1.7, in the *n*‐harmonic case from 1.5 to 2 with median again 1.7, and in the stochastic case from 0.9 to 1.6 with median 1 (Figure 8D).

### 4.5 Implications for axon diameter mapping

**Figure 9:**
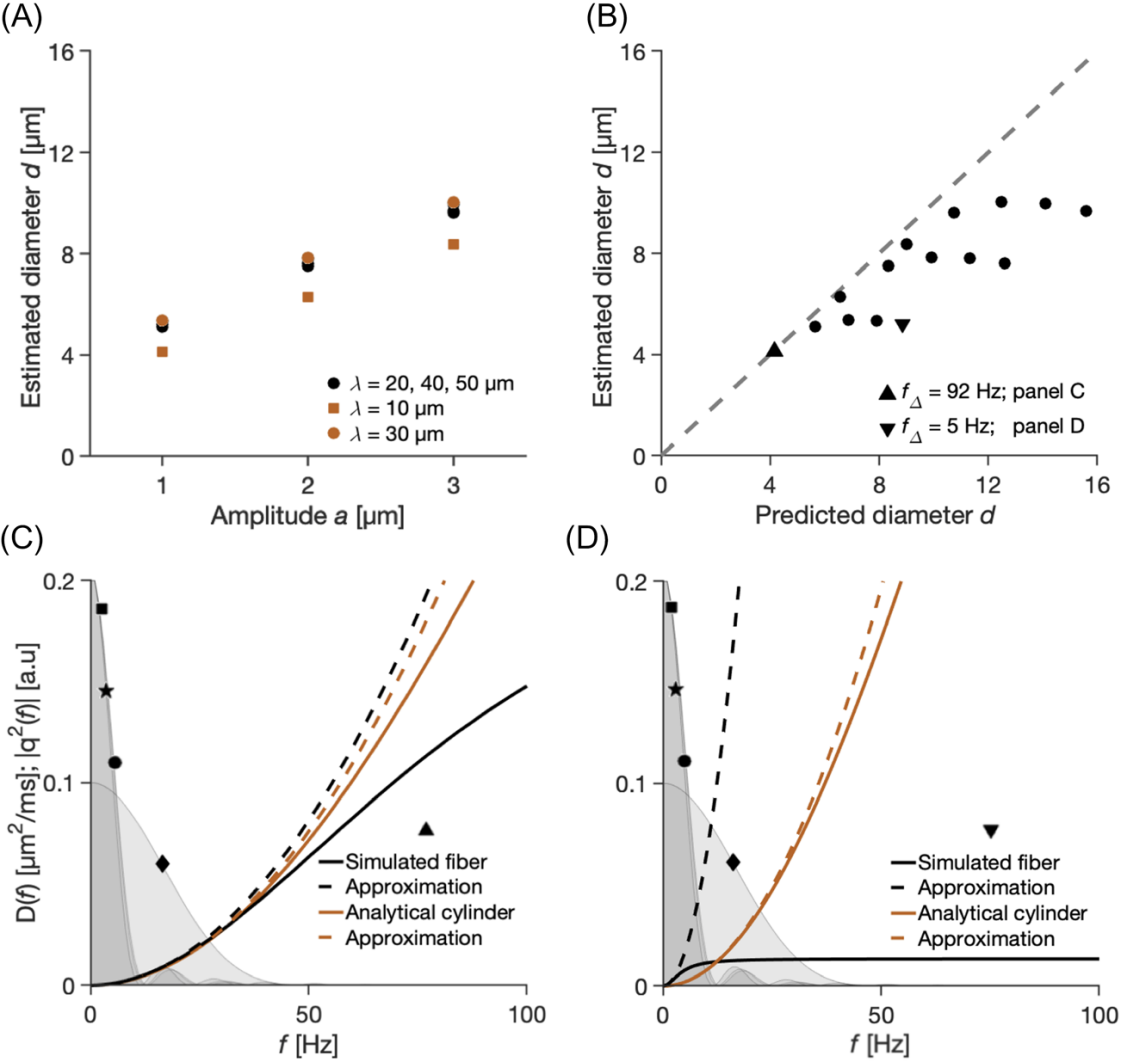
Implications for axon diameter mapping: cylinder diameter estimation. (A) Strong correlation between the undulation amplitude and the estimated diameter was found. (B) Estimated cylinder diameter versus diameters predicted based on a second‐order Taylor expansion (Equation 26). The predictions were more accurate for cases that had higher spectral widths (eg upward‐pointing triangle, corresponding to *a* = 1 μm, *λ* = 10 μm, *f*_Δ_ = 92 Hz) whereas those that had lower widths (eg downward‐pointing triangle, *a* = 1 μm, *λ* = 50 μm, *f*_Δ_ = 5 Hz) were not as well predicted. (C) The case marked with the upward‐pointing triangle from (B). the simulated diffusion spectrum (solid black line) was well approximated by its second‐order approximation (dashed black line, Equation 25) and well aligned with the diffusion spectrum of a cylinder with the estimated diameter (solid red line), which is also well approximated (dashed red line, Equation 24). Gray areas show re‐scaled encoding power spectra from Figure 6. (D) The case marked with a downward‐pointing triangle from (B), where the second‐order approximation of the simulated diffusion spectrum failed

Estimated cylinder diameters correlated with undulation amplitude. Figure 9 shows cylinder diameters estimated using a model that assumes straight cylinders from signal data generated with the 1‐harmonic case of the undulating thin fiber model. Expected values of the diameter are zero, but the estimated diameters are substantially higher even in the presence of slight deviations from straight paths (ratio between undulation amplitude and wavelength between 2 and 30 %). The relationship between estimated diameters and undulation amplitudes (Figure 9A) was not linear with respect to the wavelength *λ* because the overestimation is weakest for a wavelength of 10 μm and strongest for wavelength *λ* = 30 μm but not for *λ* = 50 μm. Hypothetically, a link between parameters of our fiber model and cylinder diameters can be established using a second‐order Taylor expansion of the diffusion spectrum (Equation 26). However, this hypothetical relationship holds only in some cases, while in others the diameter is systematically underestimated (Figure 9B). Figures 9C and 9D show the encoding spectra as well as the diffusion spectra for the cylinder and 1‐harmonic cases of our fiber model, with the difference that the hypothetical relationship between predicted and estimated cylinder diameters (Equation 26) was accurate for the undulating thin fiber used in Figure 9C (upward‐pointing triangle in Figure 9B) but not for the one in 9D (downward‐pointing triangle in Figure 9B). Comparing encoding and diffusion spectra explains why. In Figure 9C, all of the encoding power is within the quadratic part of the diffusion spectrum, where the Taylor expansion is accurate. In Figure 9D, some of the encoding power is found in the part of the spectrum where the Taylor expansion is no longer valid.

## 5 DISCUSSION

Undulating thin fibers have a diffusion time dependence that is similar to that in cylinders, at least for lower frequencies (longer diffusion times). This can lead to overestimated axon diameters when interpreting data from undulating axons using models that represent axons by straight cylinders, and this positive bias is non‐trivially associated with the undulation parameters (Figure 9). Observed effects of time‐dependent diffusion in brain white matter are subtle,^45^ and may potentially be attributed to undulation parameters rather than the axon diameter. For example, axon diameter indices of 3–12 μm were found by application of the ActiveAx model to the corpus callosum in human and monkey brains.^8^ Similar results can be explained solely by the presence of undulating but thin axons (Figure 9). Moreover, since the signal of undulating cylinders with diameters below 3 μm resembles that of undulating thin fibers (Figure 6B), a similar overestimation is expected also for undulating cylinders with diameters under this limit. That cylinders and virtually infinitesimally narrow fibers are indistinguishable at these sizes is in agreement with another study, which found the so‐called resolution limit of straight cylinders on clinical systems to be 3–5 μm.^19^ The reason why undulations can be misinterpreted as axon diameters is that undulating thin fibers and straight cylinders have similar diffusion spectra in the region sensitized by popular diffusion encoding protocols (Figure 3, Figure 5, and Table 3). Previous similar work by Nilsson et al^24^ studied the effects of axonal undulations on the diffusion propagator, and found the width of the propagator to reflect the undulation amplitude rather than the cylinder diameter. In this work, we instead investigated the velocity correlation function as an approximation of diffusion process at low *b*‐values, which allows for generalization to any gradient waveform and does not necessitate assumptions of narrow pulses (ie δ → 0).^46^ Moreover, we studied the effects of undulations from a broader perspective and found the μOD of thin fibers to be the most useful model parameter to predict features of the diffusion spectra (Figure 4). We have shown that the examined examples of diffusion spectra require only two parameters for description (spectral height and width; Figure 3)—at least if SNR ≤ 50. Further details may be accessible, however, at higher SNR. We also demonstrated the overestimation by using a popular encoding protocol from Alexander et al^8^ (Table 3).

The parameters of the undulating thin fiber model also affected the low‐frequency behavior of the diffusion spectrum, which is a feature that enables different types of structure to be grouped into a relatively few distinct structural universality classes. Specifically, the power law exponent *p* has been shown to capture long‐range spatial correlations of restrictions.^36^ Burcaw et al associated the structure of the extra‐axonal space with short‐range disorder characterized by *p* = 1, whereas the restricted diffusion in the intra‐axonal space has *p* = 2,^17^, ^18^, ^34^ as also seen in Equation 24. In our work, however, we focused on estimation of the exponent *p* from the low‐frequency part of the diffusion spectra, in frequency ranges similar to those used previously.^17^ We found that the estimated value of *p* depends on the frequency region where it is estimated (Figure 8). Experimental findings of structural disorder (*p* < 2) may thus arise from the intra‐axonal space alone, provided that axonal trajectories can be sufficiently well represented by the stochastic case of the undulating thin fiber model. Thus, the time dependence of intra‐axonal diffusion may resemble that of extra‐axonal diffusion that is characterized by *p* < 2 and possibly bias models that assume intra‐axonal diffusion to have *p* = 2.

This work highlights the importance of considering the whole three‐dimensional content of the voxel in modeling (ie non‐straight axonal trajectories as a feature in the often‐omitted third dimension) and shows that the time dependence of the intra‐axonal space cannot be fully assessed from thin cross‐sections of nerve tissue (ie from a two‐dimensional cut‐plane orthogonal to the axons).^47^ For the parameters of our fiber model, the spectral heights and widths span up to two orders of magnitude (in the 1‐harmonic case, *f*_*∆*_ = 4–92 Hz, *D*_hi_ = 0.01–1 μm^2^/ms) for biologically plausible parameters (1‐harmonic case: *a* = 1–3 μm, *λ* = 10–50 μm). Detailed histological investigations of axonal trajectories will be crucial for the prediction of the exact frequency ranges where effects of undulations could appear in practice, and thus potentially be used to falsify the model proposed herein. Such works are ongoing.^20^, ^21^

Apart from analyzing the diffusion process, we can also analyze our diffusion encoding. Here, we can define an encoding width *e*_Δ_ as the half width at half maximum of the encoding power spectrum, at least for experiments that utilize linear diffusion encoding.^48^ If the width of the encoding spectrum is substantially larger than the width of the diffusion spectrum, ie *e*_*∆*_/*f*_*∆*_ ≫ 1, this means that the spectral height is predominantly encoded into the signal. If the opposite case holds true, *e*_∆_/*f*_∆_ ≪ 1, the signal attenuation will be zero and no information about the model parameters will be encoded. This is analogous to having a cylinder diameter below the resolution limit.^19^ In the case where *e*_*∆*_/*f*_*∆*_ ≈ 1, both the spectral width and height of the diffusion spectrum are encoded into the signal. Another way to express these relationships is to consider a characteristic time for the undulating thin fiber model (1/*f*_*∆*_). If the characteristic time of the experiment (1/*e*_*∆*_) is much shorter than that of the system, we will probe mainly the μOD of the fiber segments, which determines the spectral height. In the other limit, effects of time dependence due to restriction dominate and the μOD is largely smoothed out by the diffusion. We can also consider how μOD contributes to the total orientation dispersion. In the limit, when *e*_*∆*_/*f*_*∆*_ ≪ 1, the total orientation dispersion is dominated by the macroscopic orientation dispersion. In the other extreme, when *e*_*∆*_/*f*_*∆*_ ≫ 1, both micro‐ and macroscopic orientation dispersion will determine the total orientation dispersion. Models that represent axons as sticks (thin but straight fibers) with some macroscopic orientation dispersion of the main axon directions, such as NODDI,^13^, ^14^ would thus find different values depending on the value of the ratio *e*_*∆*_/*f*_*∆*_.

We would like to highlight six limitations of this work. First, we note that the undulating thin fiber model is relevant only for the small‐diameter axons in brain white matter, and not for larger axons. For larger axons, the intra‐axonal time‐dependent diffusion would occur at similar frequencies to where undulation effects appear. Here, we set the threshold between small and large axons to 3 μm since the signals from undulating thin fibers and undulating cylinders with 3 μm diameter, ie for the mildest undulation patterns (*a*/*λ* = 2 %), are similar. This threshold also depends on the encoding scheme. Note that large axons are present only to a limited extent in the brain but are more common in the spine and for nerves outside the central nervous system. Small axons are found in the corpus callosum,^1^, ^39^, ^40^, ^41^ optic nerve,^31^, ^38^ or phrenic nerve^25^, ^43^ (Table 2). The second limitation concerns the overestimated spectral width in the 1‐harmonic case for fibers with undulation amplitudes that are large compared with the undulation wavelength (*a*/λ ≥ 30 %). This limitation may be relevant for extra‐cranial nerves and deserves further study. The third limitation is that the properties of the toy model were investigated only in the direction transversal to the main fiber direction in the two‐dimensional plane. Adding more dimensions may also help distinguishing between straight cylinders and undulating fibers. The fourth limitation is that macroscopic orientation dispersion of axons was not considered. None of the claims required the presence of macroscopic orientation dispersion, however. Nevertheless, note that the model can be applied in vivo only when effects of macroscopic orientation dispersion can be accounted for. The fifth limitation is that for the protocol optimized in reference 8 and employed in this study the diffusion spectral content above 50 Hz is not encoded into the signal. Waveforms with high‐frequency content, using eg oscillating gradient waveforms,^49^, ^50^, ^51^, ^52^, ^53^ may be required to distinguish time‐dependent diffusion effects due to cylinder‐like structures and undulating thin fibers, because only in cylinders does the spectral height reach the bulk diffusivity. The sixth limitation is that we represent axons by undulating thin fibers while omitting a wealth of other microscopic features. However, this limitation does not affect the importance of our main conclusion: that experimental data risk being misinterpreted when using the straight‐cylinder model of axons wherever axonal undulations are present. Further experimental work is needed to assess the magnitude of this risk, which could also be regarded as an opportunity to relate time‐dependent diffusion effects to the properties of axonal trajectories.

## 6 CONCLUSIONS

Fiber undulations contribute to a diffusion time dependence similar to that of straight cylinders. At biologically relevant axonal diameters and undulation parameters, we expect undulations to give rise to the most prominent time dependence. Axonal undulations can bias diameter estimation strategies if straight cylinders are used to model the intra‐axonal diffusion. At lower frequencies, undulating fibers can give rise to a time dependence substantially different from that of a cylinder with values of the power law exponent *p* below 2. Previously, such characteristics were ascribed to extra‐axonal but not intra‐axonal diffusion. Diffusion encoding with encoding power at higher frequencies than those enabled by pulsed gradient schemes might be required to resolve effects of undulations and sizes.

## FUNDING INFORMATION

This research was supported by the Swedish Research Council (grant No. 2016–03443), the Swedish Foundation for Strategic Research (grant No. AM13–0090), the Crafoord Foundation (grant No. 20170825), and Random Walk Imaging AB (grant No. MN15).

## APPENDIX A

### Generation of substrate of the *n*‐harmonic case

The amplitudes and wavelengths defining the substrate of the *n*‐harmonic case were generated from two gamma distributions. The shape (*α*) and scale (*β*) parameters of the two distributions were drawn from uniform distributions: α_*a*_~*U*(0,10), α_*λ*_~*U*(0,10), *β*_*a*_~*U*(0,3 × 10^−6^) and *β*_*λ*_~*U*(0,50 × 10^−6^). Consequently, the amplitude and wavelength distributions were formed as

(A1) $$a\;∼\;\Gamma \left(\alpha_{a},\beta_{a}\right)+1\;\mathrm{\mu m},$$

(A2) $$\lambda \;∼\;\Gamma \left(\alpha_{\lambda },\beta_{\lambda }\right)+10\;\mathrm{\mu m}.$$

To limit the range to the region where 1‐harmonic case thin fibers were investigated, both samples were then restricted to the same parameters as investigated for the 1‐harmonic case

(A3) $$a\to a\left(a>1\;\mathrm{\mu m}\;\text{and}\;a<3\;\mathrm{\mu m}\right),$$

(A4) $$\lambda \to \lambda \left(\lambda >10\;\mathrm{\mu m}\;\text{and}\;\lambda <50\;\mathrm{\mu m}\right).$$

### Computation of diffusion spectra of the *n*‐harmonic case

Unlike in the 1‐harmonic or stochastic cases, the spectra of the *n*‐harmonic case were generated synthetically. We draw *n* = 1000 samples from distributions defined through Equations A3 and A4 (ie a single pair of amplitude and wavelength corresponds to a 1‐harmonic undulating thin fiber). For each sample we predicted its spectral height (Equation 9) and width (Equation 15), assumed that the diffusion spectrum of the 1‐harmonic case is well simplified by its single‐Lorentzian approximation (Equation 22), and averaged the diffusion spectra from all 1000 samples. The validity of the spectral height and width prediction (Equation 9, Equation 15) and of the simplification by the single‐Lorentzian approximation (Equation 22) is brought up in Section 4.

### Gaussian sampling pseudo‐algorithm

- for *t* = 0 : d*t* : *t*_max_ (for each diffusion time from 0 to *t*_max_ in length of d*t* do)
  ○ *σ* = (2*D*_0_*t*)^1/2^ (define *σ* as variance of free diffusion)
  ○ for *l* = *l*_min_ : d*l* : *l*_max_ (along the whole fiber each segment of length d*l* do)
    - *∆***l** = − 4 · *σ* : d*d* : 4 · *σ* (“active region” where changes are traced)
    - *∆***y** = *y*(*l*+*∆***l** ↦ **x**) − *y*(*l* ↦ *x*) (displacement according to Equation 1)
    - **w** = exp(−*∆***l**^2^/2*σ*^2^) (define a gaussian weight)
    - 〈*∆y*^2^(*t*)〉 = 〈*∆y*^2^(*t*)〉+d*l*/(*l*_max_ − *l*_min_) ∑ **w** · *∆***y**^2^/ ∑ **w**(sum square displacements contributions weighted by a gaussian weight)

Bold letters denote vectors, multiplication of vectors is element‐wise, “↦” represents mapping from one coordinate system to another, and 〈*∆y*^2^(*t*)〉 is a symbol representing the output variable. The procedure was repeated in discrete time steps with d*t* = 100 μs for times up to *t*_max_ = 1 s in the 1‐harmonic case. In the stochastic case, we used *t*_max_ = 10 s due to slower convergence. The parameter d*d* was set to d*d* = d*l* = 0.1 μm. In the frequency domain, these numbers correspond to a maximal frequency of *f*_max_ = 5000 Hz with d*f* = 1 Hz for *t*_max_ = 1 s and to d*f* = 0.1 Hz for the case with *t*_max_ = 10 s. The computation was initiated within one period of the sine wave for the 1‐harmonic and *n*‐harmonic cases (*l*_min_ to *l*_max_). In the stochastic case, a representative sample of the whole structure must be probed for the end result to be representative (ergodic condition). An additional simulation was used to find that the sum of diffusion spectra contributions in Equation 2 converged within 30 wavelengths of the underlying harmonic sine waves (*l*_min_ to *l*_max_).

### Mean squared error

We computed the mean squared error (MSE) between the signals obtained from simulated and simplified diffusion spectra, defined as

(A5) $$\mathrm{MSE}=〈{\left(S_{L\left(f\right)}-S_{D\left(f\right)}\right)}^{2}〉.$$

Note that due to normally distributed noise *ε* at given SNR = 50 we have

(A6) $$\varepsilon ∼N\left(0,\sigma^{2}\right);E\left(\varepsilon^{2}\right)-E{\left(\varepsilon \right)}^{2}=V\left(\varepsilon \right)-0=V\left(\varepsilon \right)=\sigma^{2}$$

(A7) $$\mathrm{MSE}=〈\varepsilon^{2}〉=\sigma^{2}=4ċ10^{-4}.$$

MSE sets the limit when other changes apart from spectral width and height are detectable by the encoding protocol. In our case, if MSE is lower than 4·10^‐4^ the signal differences between the single‐Lorentzian approximation and simulated spectra are indistinguishable from noise, which means that the spectra can be described by only spectral height and width. If MSE is above 4·10^‐4^ , however, the signal differences are detectable, which may mean that more than just two parameters may be needed to characterize the spectra.
